# Kidney failure in the elderly due to hypothyroidism: a case report

**DOI:** 10.1590/S1516-31802008000500010

**Published:** 2008-09-04

**Authors:** Graziela Cristina Pichinin Ledo Silva, Jaqueline Brotto Carneiro, Cecília Carboni Tardelli, Maviane Risso, Mauricio de Miranda Ventura

**Keywords:** Aged, Kidney failure, Hypothyroidism, Hashimoto disease, Dialysis, Idoso, Falência renal, Hipotireoidismo, Doença de Hashimoto, Diálise

## Abstract

**CONTEXT:**

Hypothyroidism is more prevalent in the elderly and its symptoms can be confused with other changes due to aging. Doctors caring for the elderly need to be attentive to this diagnostic possibility. This case report case is notable not only because it presents a rare complication of hypothyroidism (kidney failure), but also because patients with chronic kidney failure of any etiology may suffer increased renal dysfunction as a result.

**CASE REPORT:**

This was a 66-year-old male outpatient with a history of generalized edema over the preceding eight years, with periods of worsening, that was intractable to treatment with diuretics. Physical examination revealed bradycardia (heart rate: 52 bpm), pallor, dry and infiltrated skin, macroglossia, edema in the lower limbs and a palpable thyroid with hard consistency. Laboratory tests showed: creatinine 3.9 mg/dl; urea 95 mg/dl; potassium 6.0 mEq/l; thyroid-stimulating hormone > 100 mUI/ml; triiodothyronine 0.01 ng/dl; free thyroxin 0.01 ng/dl; antithyroglobulin 31 IU/ml (normal values: < 40 IU/ml); antithyroperoxidase 85 IU/ml (normal values: < 15 IU/ml); creatinine clearance 30 ml/min/1.73 m^2^; and proteinuria 122 mg/24 h. After five months of treatment with thyroxin (100 mcg/day), the patient returned without any symptoms and presented the following test results: urea 48 mg/dl; creatinine 1.4 mg/dl; creatinine clearance 67 ml/min/1.73 m^2^; potassium 4.2 mEq/l; thyroid-stimulating hormone: 20.85 mUI/ml; free thyroxin: 0.71 ng/dl. Hypothyroidism alone can cause renal impairment or worsen renal function in preexisting illnesses. Its treatment can stabilize the clinical condition, or possibly improve it.

## RESUMO

**CONTEXTO:**

O hipotireoidismo tem uma prevalência elevada em idosos e sua sintomatologia pode ser confundida com alterações próprias do envelhecimento, obrigando o médico que atende o idoso sempre estar atento a essa possibilidade diagnóstica. O relato deste caso chama atenção não somente para uma rara complicação do hipotireoidismo, a insuficiência renal, como para o fato de pacientes portadores de insuficiência renal crônica de qualquer outra etiologia poderem ter piora da disfunção renal devido a esse quadro.

**RELATO DO CASO:**

Homem de 66 anos foi atendido em nosso ambulatório com história de edema generalizado há oito anos, com períodos de piora, refratário a tratamento com diuréticos. Ao exame físico encontrava-se bradicárdico (freqüência cardíaca 52 bpm), hipocorado; pele ressecada e infiltrada; macroglossia; edema de membros inferiores; tireóide palpável e de consistência endurecida. Exames laboratoriais mostraram: creatinina 3,9 mg/dl; uréia 95 mg/dl; potássio 6,0 mEq/l; hormônio estimulante da tireóide, TSH > 100 mUI/ml; T3 0,01 ng/dl; T4 0,01 ng/dl; anti-tireoglobulina 31 UI/ml (< 40 UI/ml); anti-tiroperoxidase 85 UI/ml (< 15 UI/ml); clearence de creatinina 30 ml/min/1,73 m^2^; proteinúria 122 mg/24 h. Após cinco meses de tratamento com tiroxina 100 mcg/dia o paciente retornou ao ambulatório assintomático, apresentando os seguintes exames: uréia 48 mg/dl; creatinina 1,4 mg/dl; clearence de creatinina 67 ml/min/1,73 m^2^; potássio 4,2 mEq/l; TSH 20,85 mUI/ml; T4 livre 0,71 ng/dl.

**CONCLUSÃO:**

O hipotireoidismo *per se* pode levar a insuficiência renal ou piorar a função renal em doenças preexistentes. Seu tratamento pode reverter essa condição clínica ou, pelo menos, melhorála.

## INTRODUCTION

Hypothyroidism has high prevalence among the elderly. Among individuals over 60 years old, it affects 10% of women and 2% of men.^1^

The primary form of hypothyroidism can be caused by surgery or by treatment with radioactive iodine. The idiopathic primary form of this illness is associated with circulating antithyroid antibodies,^2,3^ which can block the thyroid-stimulating hormone receptor. In some cases, drugs such as amiodarone^4^ and lithium^1^ can act like these antibodies. Hypothyroidism can coexist with other autoimmune diseases like diabetes mellitus, Addison's disease, pernicious anemia, vitiligo and chronic hepatitis.^5,6^ It may also be associated with kidney abnormalities.^7^

Symptoms such as cold intolerance, weight gain, dry skin, intestinal constipation or mental and physical slowness can be confused with the normal signs or effects of aging.^1^ Illnesses like depression and dementia can be caused or worsened by hypothyroidism. It may also be responsible for carpal tunnel syndrome, walking problems (muscle weakness), slower muscle reactions, ataxia and neuropathy. In elderly people with multiple illnesses, the use of different kinds of medication and their side effects may mimic or mask the symptoms of hypothyroidism. The laboratory test abnormalities resulting from hypothyroidism are quite varied: macrocytosis with or without anemia, elevated cholesterol and plasma triglycerides, inexplicable hyponatremia and elevation of the enzymes creatine phosphokinase and lactate dehydrogenase.^1^

Some studies have suggested that hypothyroidism should be evaluated in patients with kidney abnormalities.^8,9^ This hormonal deficiency can lead to kidney diseases: since thyroid hormones are responsible for the growth and development of the kidneys, they have an influence on substance transportation through the membrane and modify the electrolytic metabolism. This leads to deficits of renal function, associated with reductions in the cardiac output.^10,11^ It can also cause glomerulopathy (nephritic and/or nephrotic syndrome) and occur in diverse forms of chronic kidney disease, including in hemodialysis patients, thereby worsening the prognosis.^10^ Most kidney abnormalities (structural, metabolic and morphological) caused by hypothyroidism can be reverted after supplementation with thyroxin.^11^

Here, we describe the case of a patient attended at the geriatric outpatient clinic of Hospital do Servidor Público Estadual, in São Paulo, who was diagnosed as presenting renal dysfunction associated with hypothyroidism. The importance of this report lies in the high prevalence of this association, since primary hypothyroidism can occur in 9.5% of the patients with chronic renal disease, compared with a prevalence of 0.6 to 1.1% in the general population. Hence, primary hypothyroidism is often underdiagnosed.^12,13^ Moreover, the kidney disease in such patients is potentially reversible when treated appropriately.

## CASE REPORT

This was a 66-year-old male outpatient with a history of generalized edema over the preceding eight years, with periods of worsening, who was unresponsive to diuretics. Physical examination revealed bradycardia (heart rate 52 bpm), pallor, dry and infiltrated skin, macroglossia, edema in the lower limbs and an enlarged thyroid with hard consistency.

Laboratory tests showed: hemoglobin 11.2 g/dl, hematocrit 37%, mean corpuscular volume 93.2 μm^3^, creatinine 3.9 mg/dl, urea 95 mg/dl, potassium 6.0 mEq/l, creatine phosphokinase 47 U/l (normal values are from 22 to 165 U/l), thyroid-stimulating hormone > 100 mIU/ml, triiodothyronine 0.01 ng/dl, thyroxin 0.01 ng/dl, antithyroglobulin 31 IU/ml (< 40 IU/ml), antithyroperoxidase 85 IU/ml (< 15 IU/ml), creatinine clearance 30 ml/min/1.73 m^2^ and proteinuria 122 mg/24 h. The ultrasonography on the thyroid showed that its volume was at the lower limit of normality, with heterogeneous texture (thyroiditis). Scintigraphy did not define any image of the low capitation values.

After five months of treatment with thyroxin 100 mcg/day, the patient returned to the outpatient clinic without any symptoms. He presented the following test results: urea 48 mg/dl, serum creatinine 1.4 mg/dl, creatinine clearance 67 ml/min/1.73 m^2^, potassium 4.2 mEq/l, thyroid-stimulating hormone 20.85 m IU/ml and free thyroxin 0.7 ng/dl.

## DISCUSSION

Hypothyroidism in older patients is most often primary. It is caused by autoimmune disorders, previous thyroidectomy or application of treatment for hyperthyroidism. Organ-specific autoimmunity increases with advancing age and, hence, Hashimoto thyroiditis remains the main cause of thyroid failure in older patients.^14^

The different causes of hypothyroidism result in similar symptoms like bradycardia, hypertension, delayed tendinous reflex actions, cramps, increased muscle mass, weakness, dry and coarse skin, non-depressive edema, intolerance to cold and increased body weight.^14^ In most patients, the slow and gradual beginning of the illness may make diagnosis difficult. This was exactly how it occurred in the case of our patient, who was referred to us with history of long-term edema that was resistant to diuretics. Previous investigations had not led to any diagnosis of hypothyroidism.

In this case, the patient presented a very high level of thyroid-stimulating hormone and reduced levels of triiodothyronine and free thyroxin, which confirmed the previous diagnosis. The finding of antithyroid antibodies was compatible with Hashimoto thyroiditis.^14^

Hypothyroidism is often associated with kidney diseases.^15^ These renal abnormalities occur because the deficiency of thyroid hormones reduces the cardiac output. Consequently, this deficiency reduces the renal blood flow and, finally, the glomerular filtration rate.^11,16^ The result from this is an elevation in the serum levels of creatinine and a reduction in its clearance. There may also be slight proteinuria, secondary to increased capillary transudation of proteins.^8^ In serious cases, there may be acute kidney failure due to rhabdomyolysis. In our case, this was ruled out, since the level of creatine phosphokinase was normal. Likewise, hypothyroidism can also explain the deterioration in renal function among patients with chronic renal illness.

Studies that have commented on this association have suggested that thyroid hormone assays should be performed in the cases of patients whose renal function is quickly worsening. In addition, a study on patients with chronic renal failure who underwent continual outpatient peritoneal dialysis treatment showed a greater association with thyroid nodules and hypothyroidism than among control group patients. Consequently, this suggests that the presence of thyroid nodules and assays on thyroid hormone levels should be routinely investigated among chronic kidney failure patients.^15^

After a five-month period, we observed that our patient presented renal function recovery, as shown by clinical and laboratory parameters. This finding is in line with descriptions in the literature. It needs to be noted that the patient did not have any chronic illness that could have led to kidney failure and he was not using any nephrotoxic medication.

## CONCLUSION

Hypothyroidism must be suspected either as a cause of kidney disease or as a factor in the worsening of renal dysfunction in patients with chronic kidney failure. Treatment with thyroid hormones enhances renal function, thus improving the glomerular filtration rate. Assaying of thyroid-stimulating hormone levels should be considered as a screening test for patients with renal abnormalities.
